# A New Method for Preparing Mesenchymal Stem Cells and Labeling with Ferumoxytol for Cell Tracking by MRI

**DOI:** 10.1038/srep26271

**Published:** 2016-05-18

**Authors:** Li Liu, Lanya Tseng, Qing Ye, Yijen L. Wu, Daniel J. Bain, Chien Ho

**Affiliations:** 1 Department of Biological Sciences, Carnegie Mellon University, Pittsburgh, PA, USA; 2 Department of Geology and Environmental Science, University of Pittsburgh, Pittsburgh, PA, USA.

## Abstract

Mesenchymal stem cells (MSCs) are among the major stem cells used for cell therapy and regenerative medicine. *In-vivo* cell-tracking by magnetic resonance imaging (MRI) is crucial for regenerative medicine, allowing verification that the transplanted cells reach the targeted sites. Cellular MRI combined with superparamagnetic iron-oxide (SPIO) contrast agents is an effective cell-tracking method. Here, we are reporting a new “bio-mimicry” method by making use of the “*in-vivo* environment” of MSCs to prepare native MSCs, so that (i) the phagocytic activity of cultured MSCs can be recovered and expanded MSCs can be *ex-vivo* labeled with Ferumoxytol, which is currently the only FDA approved SPIO nanoparticles for human use. Using our new method, 7-day cultured MSCs regain the capability to take up Ferumoxytol and exhibit an intracellular iron concentration of 2.50 ± 0.50 pg/MSC, comparable to that obtained by using Ferumoxytol-heparin-protamine nanocomplex; and (ii) cells can be re-sized to more native size, reducing from 32.0 ± 7.2 μm to 19.5 ± 5.2 μm. Our method can be very useful for expanding MSCs and labeling with Ferumoxytol, without the need for transfection agents and/or electroporation, allowing cell-tracking by MRI in both pre-clinical and clinical studies.

Cellular magnetic resonance imaging (MRI) is an important methodology that can visualize and track cells labeled with MRI contrast agents *in vivo*^1^,^2^,^3^,^4^,^5^,^6^,^7^,^8^,^9^,^10^,^11^,^12^,^13^. Cellular MRI is essential to regenerative medicine, allowing monitoring the delivery of stem cells and/or immune cells to the desired sites in the body. The stem cells differentiate into healthy tissue that exchanges with the damaged ones^14^ and the immune cells will trigger immune responses that improve regeneration or kill tumor cells^15^,^16^,^17^. The success of cellular therapy depends on the precise dosing, timing, and delivery of the transplanted cells to the desired sites in the body. A challenge of current clinical trials on examining patients’ response to stem cell therapies is that individual variation is significant, with some patients achieving good response while some not^2^,^18^,^19^,^20^,^21^,^22^,^23^. It is possible that the transplanted cells did not engraft or survive in those that did not respond to therapy. *In-vivo* tracking of engrafted cells provides needed information, ensuring cells engraft and survive and clarifying the fate of transplanted cells, thus improving therapy accuracy and efficacy.

Mesenchymal stem cells (MSCs) are important multipotent cells and have been registered in over 360 clinical trials for at least 12 kinds of pathological conditions^14^,^24^,^25^. MRI combined with superparamagnetic iron-oxide (SPIO) contrast agents is an effective and safe non-invasive method for MSC tracking^26^,^27^,^28^. Currently, Ferumoxytol (Feraheme injection, AMAG Pharmaceuticals, MA) is the only intravenous FDA-approved SPIO nanoparticles^29^. Ferumoxytol has been approved as an iron supplement for the treatment of iron deficiency anemia in adult patients with chronic kidney disease^30^.

Ferumoxytol does not effectively label MSCs *ex vivo* (in cell culture) when used alone or in combination with protamine. The only *ex-vivo* cell-labeling method is the Ferumoxytol-heparin-protamine (HPF) nanocomplex methodology^31^. MSCs show an iron content of 2.12 ± 0.11 pg/human MSC when labeled using this method. However, the addition of transfection agents could cause undesired effects, e.g., alterations in cell biology and *in-vivo* side effects of the transfection agents. Recently, Khurana *et al.* found that MSCs are phagocytic in nature and can be labeled by an *in-vivo* cell-labeling method (i.v. injection)^32^. MSCs were labeled *in vivo* by injecting rats with a dose of 28 mg of iron per kilogram of Ferumoxytol 48 hrs before extraction, resulting in an iron content of 4.28 ± 0.19 pg/MSC. This method reduces the risk of contamination and biologic alterations of the stem cells between harvest and transplantation. However, this *in-vivo* cell-labeling method has limitations^33^: (i) This approach is not applicable to autologous MSC transplants for cell-tracking studies, because the MSC donor will have a ubiquitous presence of Ferumoxytol-labeled macrophages indiscriminant from the transplanted cells; and (ii) not applicable to methods requiring cell expansion to obtain enough labeled MSCs for clinical dosing, because cell divisions will dilute the Ferumoxytol label to below cellular MRI detection levels. An efficient *ex-vivo* labeling method for MSCs, without the need of using transfection agents and/or electroporation, is highly desired.

Khurana’s study indicated that MSCs are phagocytic in nature and can take up Ferumoxytol^32^. However, during the *ex-vivo* cell culture and expansion, MSCs become “less phagocytic” and lose the ability to take up Ferumoxytol. It is a challenge that MSC phenotype and function changes during expansion required to achieve enough cell numbers for clinical dosing^34^. Differences between minimally-cultured MSCs (2 hrs) and conventionally-cultured MSCs (7 days or longer) have been reported^35^,^36^, such as enlargement of cell size, decrease of proliferative capacity, expression of stem cell marker and chemokine receptors, expression of tumor necrosis factor-β and oncogenic transcription factor c-Myc, and loss of self-renewal capacity and multipotency. Notably, cell size has been found to be an important characteristic of MSCs^36^,^37^,^38^,^39^. Smaller MSCs exhibit better self-renewal and differentiation capacity and bigger MSCs show signs of senescence^39^,^40^. Recently, it has been found that the gene expression of STRO-1, TWIST-1 and DERMO-1 are correlated with the cell size and potency of MSCs^41^. Scientists are trying to identify the methodologies to enable prolonged expansion and rejuvenation for MSCs^36^,^42^.

We have two aims in this study: (i) to investigate the “changes”, e.g., phagocytic capability, of MSCs during *ex-vivo* culture and expansion; and (ii) to recover the “changes” of MSCs after *ex-vivo* expansion, so that MSCs can be better prepared *ex vivo* and expanded MSCs can be more “native”. Our hypothesis is that the “*in-vivo* cellular environment” is important for MSC functions and can recover the “changes” of the *ex-vivo* expanded MSCs. If we can recover the phagocytic capability of expanded MSCs, MSCs can be labeled with Ferumoxytol in cell culture, without the need for transfection agents and/or electroporation. It can also be very useful for cell-tracking by MRI in both clinical and pre-clinical studies.

## Results

### Cell Labeling, Characterization, and Viability

**Figure 1:**
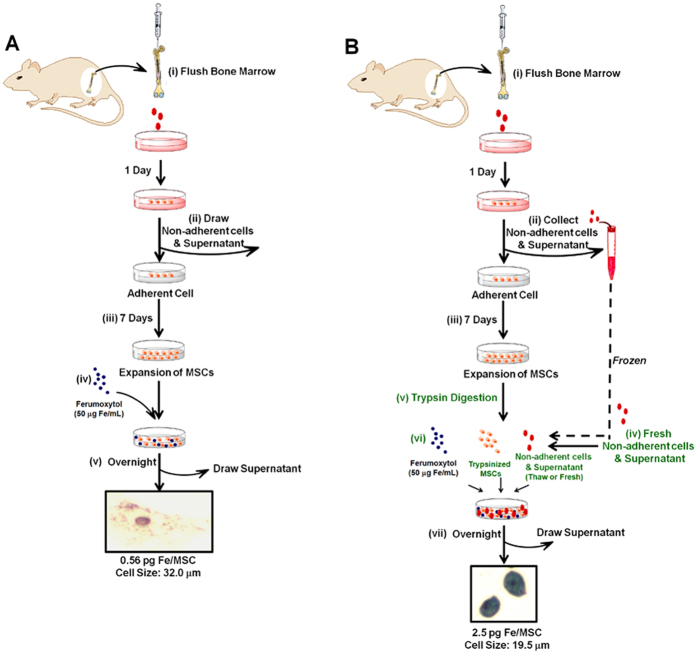
Flow chart depicting (**A**) traditional method and (**B**) new “bio-mimicry” method for labeling bone marrow MSCs with Ferumoxytol.

**Figure 2:**
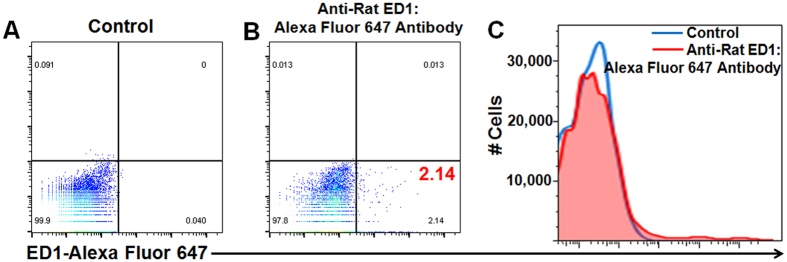
Flow cytometry analysis of anti-ED1 staining of the MSCs harvested after labeling with Ferumoxytol through our new “bio-mimicry” method: (**A**) MSCs stained with isotype control; (**B**) MSCs stained with mouse anti-rat ED1:Alexa Fluor 647 antibody; and (**C**) histogram of the data shown in (**A**,**B**).

The detailed procedures of the traditional method (Fig. 1A) and our new “bio-mimicry” method (Fig. 1B) are described in **Materials and Methods.** The purity and phenotype of MSCs prepared through this method have been investigated in our previous publication^43^. Briefly, MSCs were stained with CD166, CD105, CD44, CD29, MHC-I, and CD34. Flow cytometry results show that the purity of MSCs was 92–95%. We also tested the presence of monocytes/macrophages in the MSCs by staining with ED1, which is the rat homologue of human CD68. Figure 2 shows a representative figure of the flow cytometry analysis of the MSCs harvested after labeling with Ferumoxytol through our new “bio-mimicry” method. About 2% of the cells are ED1-positive cells. The Ferumoxytol-labeled MSCs from our new method show over 95% viability. We have not observed cell aggregation after Ferumoxytol labeling.

### Decreased Phagocytic Activity and Increased Cell Size of MSCs after Day 4 of Culture

**Figure 3:**
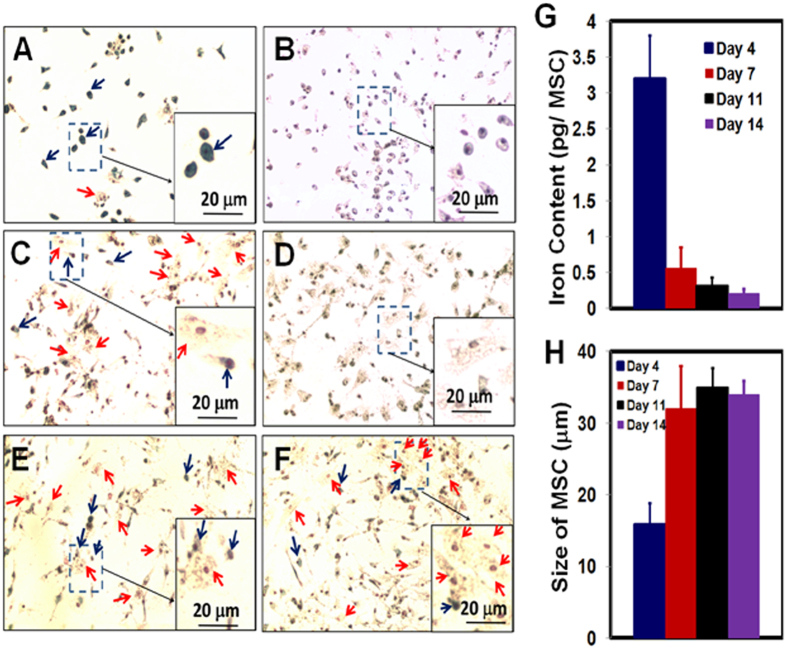
Perls’ Prussian blue iron staining of MSCs co-incubated with Ferumoxytol (**A**,**C**,**E**,**F**) and control MSCs (absence of Ferumoxytol, (**B**,**D)**). MSCs are from day 4 (**A**,**B**), day 7 (**C**,**D**), day 11 (**E**), and day 14 (**F**) of culture. An enlarged view of MSCs is shown as an example in each figure. Blue arrows are pointing Prussian blue positive cells. Red arrows are showing Prussian blue negative cells. The changes of iron concentrations and cell sizes are summarized in (**G**,**H)**, respectively.

In this experiment, MSCs were labeled by the traditional method (Fig. 1A). On days 4, 7, 11, and 14 of MSC culture, Ferumoxytol (50 μg Fe/mL) was added to the culture medium (Fig. 3A,C,E,F, respectively). After overnight co-incubation, the labeling efficiency was determined by Perl’s Prussian blue iron staining. Figure 3A shows that the MSCs, on day 4 of culture, still exhibit great phagocytic activity and can be labeled with Ferumoxytol efficiently. As indicated by the blue arrows in Fig. 3A, the majority of the MSCs are Prussian blue positive and the shape of these cells is small and round. Only a small portion of the MSCs are Prussian blue negative (pointed by red arrows) and the shape of these cells is bigger and flat. On days 7, 11, and 14 of culture (Fig. 3C,E,F), the majority of MSCs cannot be labeled with Ferumoxytol (red arrows) and the shape of these cells is big and flat. Only a small portion of small and round cells can be labeled with Ferumoxytol (blue arrows). Thus, after day 4 of culture (days 7, 11, and 14), the labeling efficiency with Ferumoxytol decreases significantly.

Figure 3B,D show the results of Prussian blue staining of MSCs, on days 4 and 7 of culture, with no addition of Ferumoytol. Since there is a small amount of iron in the MSCs in nature, some cells show a light blue color (Fig. 3B). Thus, the strong blue color of iron staining in Fig. 3A is the result of the uptake of Ferumoxytol by MSCs.

The results of Perl’s Prussian blue iron staining are confirmed by iron concentration measurement by inductively coupled plasma-mass spectrometry (ICP-MS) (Fig. 3G). When Ferumoxytol was co-incubated with MSCs on day 4 of culture, the labeled MSCs exhibit an intracellular iron concentration of 3.2 ± 0.6 pg/MSC. When Ferumoxytol was co-incubated with MSCs on days 7, 11, and 14 of culture, the resulted iron concentrations are 0.56 ± 0.29, 0.32 ± 0.11, and 0.21 ± 0.06 pg/MSC, respectively.

As summarized in Fig. 3H, we have also observed that the cell size of MSCs increases beginning day 7 of culture. The MSCs on day 4 are mostly small and round (Fig. 3A,B). The average size of these cells is 17.2 ± 1.9 μm. The MSCs on days 7, 11, and 14 are large and flat (Fig. 3C–F). The size of MSCs increases to over 30 μm since day 7.

### New “Bio-mimicry” Method: Recover Phagocytic Activity and Re-size MSCs

**Figure 4:**
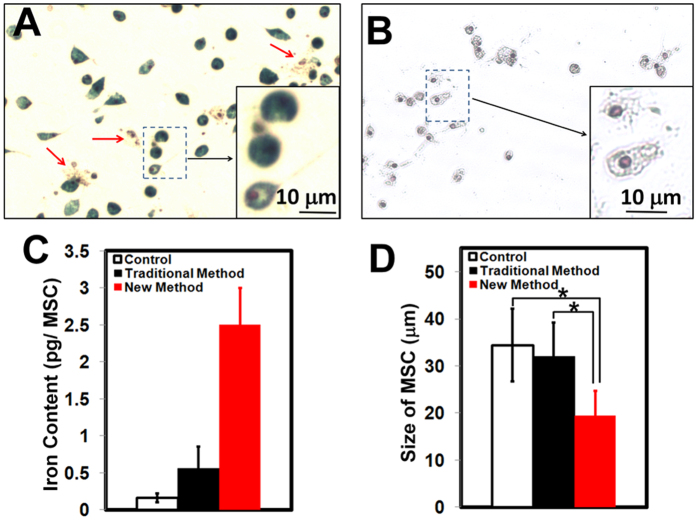
“Bio-mimicry” method can increase the labeling of MSCs by Ferumoxytol and decrease size of MSCs on day 7 of culture. (**A**) Prussian blue staining of MSCs after Ferumoxytol labeling through the “bio-mimicry” method. Red arrows are pointing Prussian blue negative cells. (**B**) Prussian blue staining of MSCs from the “bio-mimicry” labeling method, but absence of Ferumoxytol. Summary of iron concentration (**C**) and cell size (**D**) of MSCs: normal MSC, MSCs from the traditional labeling method, and MSCs from “bio-mimicry” labeling method. ^*^p < 0.0001.

In this experiment, MSCs were labeled by our new “bio-mimicry” method (Fig. 1B). Freshly prepared non-adherent cells and supernatant is used to “mimic” the “*in-vivo* environment” of MSCs (Fig. 1B, step **iv**). Prussian blue iron staining of labeled MSCs from our new method after step **vii** is shown in Fig. 4A. Approximately 90% of MSCs show positive blue color after staining and only a small fraction show Prussian blue negative (as indicated by red arrows). As a comparison, Fig. 3C shows the iron staining of MSCs on day 7 of culture labeled by the traditional method, in which most of the MSCs are Prussian blue staining negative and only a small fraction show blue color (pointed by blue arrows). Iron concentration measurement by ICP-MS confirms this result (Fig. 4C). By the traditional method, the intracellular iron concentration is 0.56 ± 0.29 pg Fe/MSC. By our new method, the iron concentration increases to 2.50 ± 0.50 pg Fe/MSC, which is comparable to that obtained with the use of HPF nanocomplexes (2.12 ± 0.11 pg Fe/human MSC)^31^. Thus, our new method recovers the phagocytic activity of MSCs on day 7 of culture.

Our “bio-mimicry” method also can re-size MSCs. After co-incubation with the non-adherent cells and supernatant overnight (Fig. 1B, step **vi** and **vii**), the size of labeled MSCs decreases to 19.5 ± 5.2 μm (Fig. 4A,B,D), which is significantly smaller than the usual size of MSCs on day 7 of culture (32.0 ± 7.2 μm, Fig. 3C,D). As shown in Fig. 4A, the majority of MSCs are small and Prussian blue staining positive. Only a small portion of the cells, as indicated by red arrows, are large and Prussian blue staining negative. Fig. 4B shows the Prussian blue iron staining of MSCs from this new method, with no addition of Ferumoxytol. Thus, the decrease in cell size is not caused by Ferumoxytol and the strong blue color, shown in Fig. 4A, is caused by cellular uptake of Ferumoxytol. Fig. 4D is a summary of the size of MSCs on day 7 of culture: normal MSCs, MSCs from the traditional labeling method, and MSCs from the “bio-mimicry” method.

### Using Frozen Non-adherent Cells and Supernatant

In an attempt to avoid the need for another bone marrow flush or aspiration, we froze the non-adherent cells and the supernatant from step (**ii**) (Fig. 1B) at −80 °C and use the thawed supernatant in step (**vi**) (Fig. 1B). We have found that the fresh supernatant (step **iv,** Fig. 1B) works more efficiently than the frozen supernatant. While the intracellular iron concentration is 2.50 ± 0.50 pg/MSC when fresh supernatant is used (Fig. 4), the intracellular iron concentration is 0.91 ± 0.22 pg/MSC when the thawed supernatant is used.

### MR Microscopy (MRM) and Transmission Electron Microscopy (TEM) of Labeled MSCs

**Figure 5:**
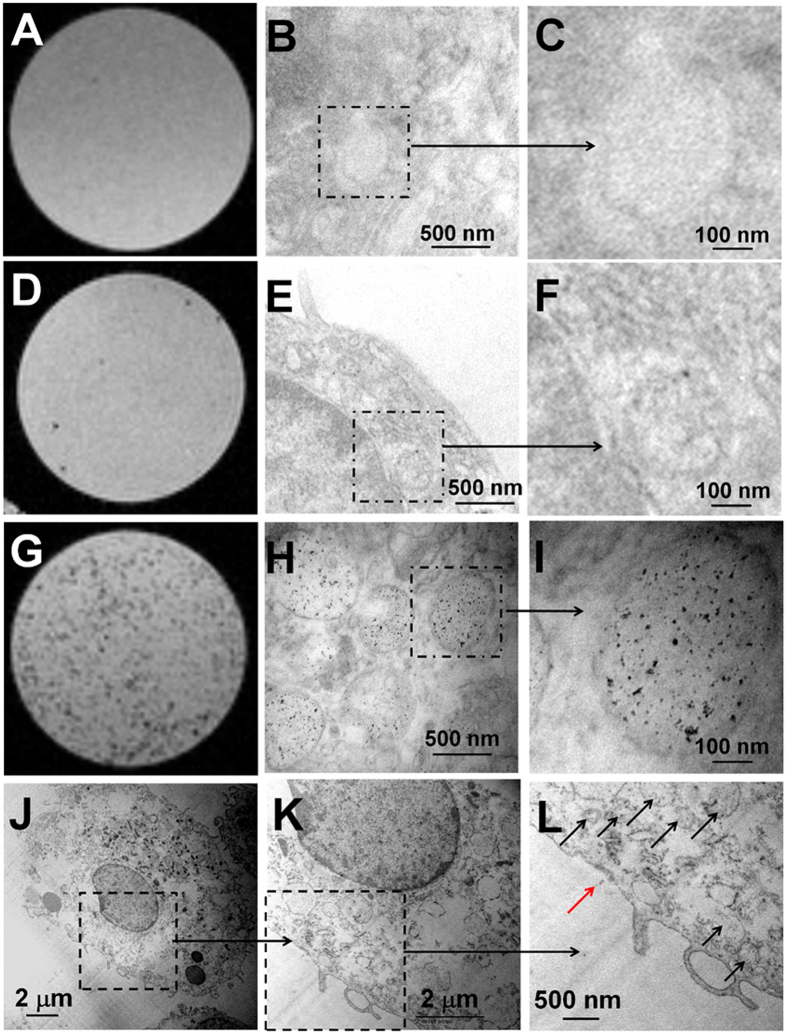
MR microscopic images (**A**,**D**,**G**) and TEM images (**B**,**C**,**E**,**F**,**H**–**L**) of MSCs: normal MSC (**A–C**), MSCs from the traditional labeling method (**D–F**), and MSCs from “bio-mimicry” labeling method (**G–L**). (**C**,**F**,**I**) are examples of enlarged views of (**B**,**E**,**H)**. (**K**,**L**) are examples of enlarged views of (**J**).

Using the tradition labeling method, the number of Ferumoxytol nanoparticles inside a MSC is very limited as compared to using our “bio-mimicry” labeling method shown in MRM images and TEM images (Fig. 5D–F vs G–L). MRM images show that there are many more hypointense spots from the gelatin phantom of labeled MSCs using our new method (Fig. 5G) than using the traditional method (Fig. 5D). Very few hypointense spots could be clusters of the labeled MSCs as seen from the high-resolution MRM images of the gelatin phantom made from the MSCs labeled by the traditional method (Fig. 5D). This result is consistent with previous reports that Ferumoxytol alone cannot label MSCs efficiently^31^,^32^. TEM images reveal that the majority of Ferumoxytol nanoparticles are localized in the vacuoles of MSCs (Fig. 5H–L). We have observed a lot of iron-oxide nanoparticles in the cytoplasm of MSCs (black arrows, Fig. 5L), but few (red arrow, Fig. 5L) on the cell membrane. The iron core diameter of Ferumoxytol appears to be ~5–8 nm in size.

### Non-adherent Cells are the Working Component

**Figure 6:**
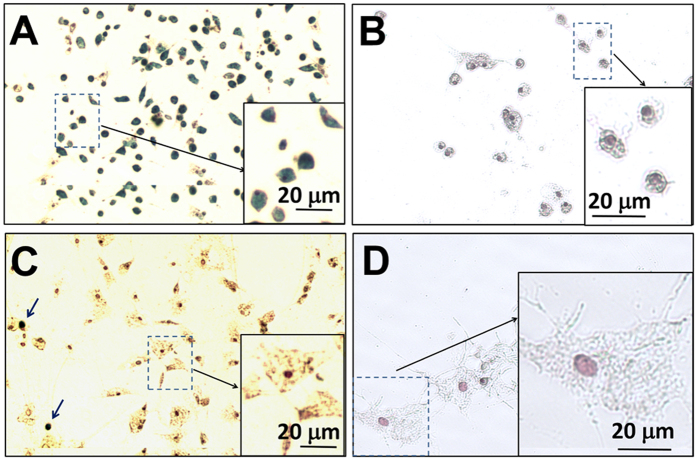
Prussian blue iron staining of MSCs after co-incubation with non-adherent cells (**A**,**B**) and supernatant liquid (**C**,**D**). (**A**,**C**) Ferumoxytol was added to cell culture. Original magnification: 100×. (**B**,**D**) Control MSCs with no addition of Ferumoxytol. Original magnification: 200×.

A major difference between the “bio-mimicry’ method and the traditional method is the addition of an “*in-vivo* environment” resulting from step (**iv**) of Fig. 1B. There are two major components in this “*in-vivo* environment” mixture: supernatant liquid and non-adherent cells. Supernatant liquid contains many cell signaling molecules and non-adherent cells contain many types of cells. We did an experiment to determine which component is the major component that changes the phagoctytic activity and size of MSCs. Non-adherent cells were separated from supernatant by centrifugation at 300 g for 5 min. Then, either non-adherent cells (Fig. 6A,B) or supernatant (Fig. 6C,D) was applied for cell labeling. As shown in Fig. 6A,B, it is the non-adherent cells that increase the labeling of MSCs (Fig. 6A) and reduce the size of MSCs (Fig. 6A,B). When the supernatant liquid is applied, most of the MSCs cannot be labeled with Ferumoxytol efficiently (Fig. 6C) and the shape of MSCs is large and flat (Fig. 6C,D). Only a small portion of MSCs can be labeled with Ferumoxytol (Fig. 6C, blue arrows) and, interestingly, the shape of these cells are small and round. Fig. 6B,D are controls: no Ferumoxytol was added. Thus, the strong blue color, as shown in Fig. 6A, is caused by cellular uptake of Ferumoxytol, and the changes of cell size and shape are not caused by Ferumoxytol.

## Discussion

*In-vivo* cell tracking by MRI offers great potential for cellular therapies and regenerative medicine. To reach this potential, new MRI techniques, new classes of contrast agents approved by FDA, and new cell labeling methodologies are required. Our laboratory has studied the labeling of MSCs with several types of superparamagnetic iron-oxide based MRI contrast agents, e.g., dextran-coated ultra-small SPIO particle (~30 nm in diameter), polyethylene glycol coated SPIO particle (~60 nm in diameter), dextran coated Feridex (120–180 nm in diameter), and styrene-divinyl benzene polymer-coated micro-sized iron-oxide particle (~1 micron in diameter)^43^. MSCs have generated great interest for regenerative medicine^25^,^44^ and many MSC trials have been carried out during the past 8 years^14^,^24^. The success of cell therapy depends on a number of factors, e.g., precise dosing, timing, and delivery of the cells to the desired sites. Tracking engrafted cells in an intact living organism is crucial. Several clinical trials for cellular therapy have incorporated MRI cell tracking into the trial protocol, using the only FDA approved SPIO particle, Ferumoxytol, as the contrast agent (https://clinicaltrials.gov). Ferumoxytol has a carboxymethylated dextran coating and a particle size of 17–31 nm in diameter, with a 6-nm iron oxide crystal core. The longitudinal *r*_*1*_and transverse *r*_*2*_ relaxivities of Ferumoxytol are 15 and 89 mM ^−1^s^−1^, respectively^45^.

Notably, Ferumoxytol does not effectively label MSCs *ex vivo* (in cell culture) without transfection agents^31^. Recently, it has been found that MSCs are phagocytic in nature and can be labeled with Ferumoxytol *in vivo* (i.v. injection)^32^. Thus, the phagocytic activity of MSCs decreases during *ex-vivo* culture. The goal of this study is to produce “more native” MSCs and recover their phagocytic activity after cell culture, so that MSCs can be expanded, then labeled with Ferumoxytol *ex vivo*, with no need of transfection agents and/or electroporation.

First, we have found that MSCs gradually lose the capability to take up Ferumoxytol during the *ex-vivo* cell culture and expansion (Fig. 3). The “*in-vivo* environment”, as applied in our “bio-mimicry” method, can recover the phagocytic activity of MSCs (Figs 4, 5, 6). By this “bio-mimicry” method, the iron concentration increases to 2.50 ± 0.50 pg Fe/MSC, similar to that obtained with the use of HPF nanocomplexes (2.12 ± 0.11 pg/MSC)^31^. The advantage of HPF nanocomplex methodology is that it is a general one and can be used to label different types of cells with Ferumoxytol, e.g., MSCs, neural stem cells, hematopoietic stem cells, T-cells, and monocytes, thus very important for cell tracking studies by MRI. The advantage of this “bio-mimicry” method is that it does not need the transfection agents, thus reducing the manipulation to the cells and the administration of transfection agents into the patients. It has been recommended by the FDA that “minimally manipulated” cells be used for human clinical trials.

Second, we have found that the phagocytic activity and size of MSCs are related. Morphologically, small and round MSCs show greater capability of uptake Ferumoxytol than big and flat cells. The relationship between cell size, morphology, and senescence of MSCs has been studied^36^,^37^,^38^,^39^,^40^,^41^. Small MSCs show high capacity in growth and differentiation, while larger cells take longer to undergo rounds of division (senescent)^39^,^40^,^41^. We have observed a gradually increase of the cell size during *ex-vivo* cell culture and expansion, which is consistent with previous reports^36^,^39^.

Third, the *in-vivo* signaling events or environments is important for MSCs, not only for labeling with Ferumoxytol, but also for giving more “native” MSCs as indicated by cell size. The importance of this “*in-vivo* environment” mixture has also been indicated by another study^42^. Zhang *et al.*^42^ have investigated and compared several conditions to isolate and culture MSCs: untreated whole bone marrow adherent culture, 3 volumes of red blood cells (RBC) lysed with ammonium chloride, 6 volumes of RBC lysed with ammonium chloride, and Ficoll density gradient centrifugation. As found by Zhang *et al.*^42^, the untreated whole bone marrow adherent cultures, which contain the MSC “*in-vivo* environment” as described in this manuscript, are best for MSC isolation and culture and the resulting cells have the strongest proliferation capacity. The Ficoll purified cultures, which eliminate most of the “*in-vivo* environment”, give the weakest proliferation capacity. There are many components in the supernatant solution of the “*in-vivo* environment”, e.g., ions, chemokines/cytokines. There are around 20 types of cells in the bone marrow^46^. In this study, we have found that the “cell partners” in the “*in-vivo* environment” mixture are critical for re-sizing MSCs and the phagocytic activity of MSCs (Fig. 6). The cell-cell interaction is critical for *ex-vivo* cell culture and labeling of MSCs with Ferumoxytol. Fishing out the major types of cells that affect MSC phagocytic activity and size will be next step of this work.

As the result, by our “bio-mimicry” method, MSCs can be labeled with Ferumoxytol with intracellular iron content of 2.50 ± 0.50 pg Fe/MSC, which is comparable to that obtained with the use of HPF nanocomplexes (2.12 ± 0.11 pg/human MSC)^31^. Previous studies^31^,^43^ have shown that this iron content will not affect the proliferation, function, and differentiation of Ferumoxytol-labeled MSCs. Thu *et al.*^31^ have found that 1 × 10^3^ HPF-labeled human MSCs with iron content of 2.12 ± 0.11 pg/MSC can be visualized in the mouse brain by *in-vivo* MRI using a 3-T clinical scanner through the T2*-weighted technique. Khurana *et al.*^32^ have reported that 1 × 10^6^ Ferumoxytol-labeled MSCs with iron content of 4.28 ± 0.19 pg/MSC can be tracked for 4 weeks in osteochondral defects using a rat model with a GE 7-T animal MRI.

In conclusion, we have three major findings: (i) the “*in-vivo* environment” in our “bio-mimicry” method can recover the phagocytic activity of cultured MSCs, so that MSCs can be labeled by Ferumoxytol (2.50 ± 0.50 pg Fe/MSC) after cell culture and expansion, with no need of transfection agents and/or electroporation; (ii) the “*in-vivo* environment” can re-size the cultured MSCs (32.0 ± 7.2 μm vs 19.5 ± 5.2 μm); (iii) morphologically, small and round MSCs show greater phagocytosis of Ferumoxytol than large and flat MSCs.

## Materials and Methods

### Materials and Animals

Male Brown Norway (BN) rats obtained from Harlan (Indianapolis, IN) with body weights between 250 and 280 g were used in this research. All experiments involving animal subjects were approved by the Institutional Animal Care and Use Committee of Carnegie Mellon University. All the animal experiments were performed in accordance with the guidelines and regulations from the Institutional Animal Care and Use Committee of Carnegie Mellon University. Animal care was provided in accordance with the Guide for the Care and Use of Laboratory Animals.

### MSCs Preparation

A “direct adherence” method as described in previous publications from our laboratory^43^ and others^12^,^32^,^42^,^47^,^48^, was used to prepare MSCs with some modifications. The procedures are shown in Fig. 1A,B, steps (**i**) to (**iii**). Briefly, (**i**) bone marrow cells were flushed from BN rat femurs and tibias with 50 mL of Dulbecco’s modified Eagle’s medium (DMEM, Mediatech) supplemented with 10% heat-inactivated fetal bovine serum (FBS) (Invitrogen), 100 U/mL penicillin, 100 μg/mL streptomycin, and 2 mM glutamine. About 300 × 10^6^ cells can be collected from each rat. Cells were plated in 12 plates (10-cm) in the above medium and incubated at 37 °C with 5% CO_2_ in a humidified (85% humidity) cell culture incubator for 24 hr. (**ii**) After 24 hr of incubation, non-adherent cells and supernatant were removed. (**iii**) MSCs were cultured and expanded. The above medium was added and replaced every 72 hr.

### Traditional *Ex-vivo* Method for Labeling MSCs with Ferumoxytol

The procedure of traditional labeling method is shown in Fig. 1A: (**iv**) after MSC culture and expansion (**i–iii**), add Ferumoxytol (50 μg Fe/mL) to the cell culture medium; (**v**) allow the cells to co-incubate overnight, followed by washing with phosphate-buffered saline (PBS) and harvesting by trypsin-EDTA digestion.

### New *Ex-vivo* Method for Labeling MSCs with Ferumoxytol

The procedure of our new bio-mimicry method is shown in Fig. 1B: (**iv**) after MSC preparation and expansion (steps **i**–**iii**) and one day before labeling, prepare fresh non-adherent cells and supernatant by repeating step (**i**), using another BN rat, and allow these bone marrow cells incubation and attaching for 24 hr; (**v**) trypsin-EDTA digest MSCs from step (**iii**) and wash MSCs with PBS; (**vi**) add Ferumoxytol (50 μg Fe/mL) to the cells and add the non-adherent cells and supernatant from step (**iv**); and (**vii**) allow the cells to incubate overnight. Steps **iv**, **v**, and **vi** are different from the traditional *ex-vivo* method, as shown in Fig. 1A.

### ED1 (CD68) Staining and Flow Cytometry

After MSCs were labeled with Ferumoxytol (Fig. 1B, step **vii**) and harvested, MSCs were washed with PBS twice and stained with anti-ED1 antibody. ED1 is the rat homologue of human CD68. As described in our previous work^3^,^49^, mouse anti-rat ED1:Alexa Fluor 647 antibody (AbD SeroTec, Oxford, UK) was used to stain monocytes/macrophages and BUF09 (AbD SeroTec) was used as a permeabilization reagent for ED1 detection. Mouse IgG1:Alexa Fluor 647 (BioLegend) was used as our isotype control. Flow cytometry was performed on a FACSVantage (Becton Dickinson, Franklin Lakes, NJ). The data were processed with the use of FlowJo software (TreeStar, Ashland, OR).

### Viability

The viability of Ferumoxytol-labeled and unlabeled (control) cells were evaluated by Trypan blue exclusion assay (Sigma).

### Perls’ Prussian Blue Iron Staining

Prussian blue staining was performed to test the presence of iron in the labeled cells using an Iron Stain Kit (Sigma-Aldrich), according to the supplier’s protocol. Staining results were examined by light microscopy and photomicrographs were taken using a Moticam 2300 camera mounted on a Nikon Diaphot microscope with Mtic Images Plus 2.0 software.

### Measurement of Cell Size

Photomicrographs were taken using a Moticam 2300 camera mounted on a Nikon Diaphot microscope. 6 fields of view were taken randomly for each plate of cells. 6 plates of cells from 3 individual experiments for each grow and labeling condition were used. The size of each cell was measured manually using Mtic Images Plus 2.0 software.

### ICP-MS for Iron Content

2 × 10^6^ cells (labeled by our new method, labeled by traditional method, or unlabeled) were decomposed in 70% nitric acid (250 μL) at 60 °C overnight. The samples were centrifuged at 400 g for 15 min and the supernatant was collected in a separate test tube. Samples were diluted and analyzed for iron concentrations by ICP-MS (NexION 300X, PerkinElmer Inc.) as described previously^49^. ^57^Fe isotope counts were used to determine the Fe content.

### MRM

A total of 2 × 10^5 ^MSCs (labeled by our new method, labeled by the traditional method, or unlabeled) were suspended in 0.2 mL 1% agarose gel for MRI with a Bruker 11.7-T scanner, equipped with a Micro 2.5 gradient set (Biospec, Avance-DBX, Bruker). High-resolution 3D images were acquired with the following parameters: repetition time (TR) = 2500 ms; echo time (TE) = 7.5 ms; number of averages (NA) = 4; and isotropic resolution = 55 μm.

### TEM

Cells were fixed in 2% paraformaldehyde (PFA) buffered with PBS at 4 °C overnight, followed by washing with PBS twice. Cells were then fixed in 1% OsO4 buffered with PBS for 1 hr and then washed three times with distilled water. The samples were dehydrated using a gradient series of ethanol and embedded in an Epon-Araldite resin. 100-nm sections were cut using a DDK diamond knife on a Reichert-Jung Ultracut-E ultramicrotome (Leica, Wetzlar, Germany). The sections were not stained with lead citrate or uranyl acetate. The sections were then mounted onto copper grids and imaged on a Hitachi 7100 transmission electron microscope (Pleasanton, CA) operated at 75 kv. Digital images were obtained using an AMT Advantage 10 CCD Camera System (Advanced Microscopy Techniques Corporation, Danvers, MA) and NIH Image software (Bethesda, MD).

### Statistical Analysis

Statistical analysis was carried out with Student’s *t* test. A *p* value <0.05 was considered statistically significant.

## Additional Information

**How to cite this article**: Liu, L. *et al.* A New Method for Preparing Mesenchymal Stem Cells and Labeling with Ferumoxytol for Cell Tracking by MRI. *Sci. Rep.* **6**, 26271; doi: 10.1038/srep26271 (2016).
